# Steps taken by the World Health Organization African Region Member States to standardise herbal medicines: a literature review

**DOI:** 10.7189/jogh.15.04265

**Published:** 2025-10-17

**Authors:** Dennis Kithinji, Ossy MJ Kasilo, Olobayo Kunle, Philippe Doo-Kingue, Mohamed Ismail, Kizito Nsarhaza

**Affiliations:** 1 Meru University of Science and Technology, Meru, Kenya; 2 Medright Consulting LTD, Maua, Kenya; 3 Traditional, Complementary and Integrative Healthcare Coalition, Geneva, Switzerland; 4 National Centre for Naturopathic Medicine, Faculty of Health Science, Southern Cross University, Lismore, Australia; 5 National Institute for Pharmaceutical Research and Development, Abuja, Nigeria; 6 World Health Organization Regional Office for Africa, Brazzaville, Congo

## Abstract

**Background:**

Standardised herbal products present a solution to the high burden of diseases and inadequate access to drugs in Africa. This literature review explores the steps that the Member States of the World Health Organization Regional Office for Africa have taken to standardise herbal medicines.

**Methods:**

We retrieved publications from Google and Google Scholar search engines and through published and non-published information physically available at the WHO AFRO. The search terms to identify the publications were ‘herbal medicine’ OR ‘traditional medicine’ AND ‘member state’ between 2013 and 2023, where ‘member state’ stood for the individual names of the World Health Organization African region member states. We extracted information on the regulation, established structures in the ministry of health, registration systems, and research and training facilities on herbal medicines from the retrieved publications.

**Results:**

Member States have taken significant steps toward standardising herbal medicines through policymaking and research, such as clarifying the necessary regulatory framework; establishing a department, division, national office, or expert committee in their ministries of health; instituting a system for registering herbal medicines; and establishing research and training facilities for herbal medicine. However, only a few Member States have implemented all four aspects of herbal medicine standardisation and registration processes, with several of them lacking dedicated registration systems.

**Conclusions:**

The WHO AFRO Member States are steadily establishing systems for the standardisation and registration of herbal medicines. Those that have made strides in specific aspects of standardisation and registration, as highlighted in the country-by-country analysis, should serve as benchmarks to others.

Africa, which bears approximately a quarter of the global disease burden, is struggling with an increasing incidence of non-communicable diseases while still tackling the challenge of infectious diseases [1,2]. Although COVID-19 did not have the anticipated impact on the continent, Africa is still not sufficiently ready to counter both burdens simultaneously. Pharmaceutical manufacturing in Africa uses expensive imported raw materials [3], with research and development (R&D) remaining poorly established, forcing countries to import medicines. In fact, only 3% of global drug supply is produced in Africa; this dependence on imports makes several drugs unaffordable to its populations, which in turn leads to the introduction of substandard and counterfeit medical products into its markets [4].

Traditional medicine (TM), meanwhile, is easily available, acceptable to the African population, and affordable, making it a potential solution to these supply challenges. Research has shown that reliance on herbal medicines increased during the COVID-19 pandemic, as countries manufacturing essential pharmaceuticals on which the African continent relied restricted exports to meet their domestic needs [3,4].

Herbal medicines based on traditional knowledge and diverse flora present an opportunity for the production of affordable medicines within Africa [5]. However, the effective use of herbal medicines would require their integration into national health care systems [6]. Such efforts are often derailed by the scepticism of conventional health care practitioners, which could be alleviated through proper registration and regulation of herbal medicines [6]. The standardisation of herbal medicines in Africa would therefore require scientific evidence on their quality, safety, and efficacy, as well as adherence to internationally recognised protocols for their eventual approval and registration.

To improve the capacity for the standardisation and institutionalisation of traditional African medicine (TAM) within the African Region, the World Health Organization (WHO) Regional Office for Africa (AFRO) developed three regional strategies: ‘Promoting the Role of Traditional Medicine in Health Systems: A Strategy for the African Region (2000–2010)’ [7]; ‘Enhancing the Role of TM in Health Systems: A Strategy for the African Region (2013–2023)’ [8]; and ‘Tools for Institutionalizing TM in Health Systems in the African Region (2004)’ [9]. The strategies call for biomedical and operational research on TAMs, the formulation of policies for their standardisation and the development of frameworks for their integration into health systems and services, and the improvement of local industries to manufacture herbal medicines [7,8]. These strategies comprise guidelines for national policies, model legal frameworks for the practice of TAM, model codes of ethics and practice, and guidelines for the formulation of a national master plan (also called national strategic plan) for the development of TAMs [9].

Aside from these strategies, the WHO AFRO developed the ‘Regional Framework for the Regulation of TM Practitioners, Practices and Products’ in 2016 [10]. The ‘Guidelines for the Clinical Study of TMs in the WHO African Region’, published in 2004, contain protocols that countries have used for conducting clinical trials of traditional medicines for the treatment of malaria, opportunistic infections related to HIV/AIDS, diabetes, hypertension, and sickle-cell disease [11]. The WHO also developed ‘Guidelines for the Registration of TMs in the African Region in 2004’ [11], which have accelerated the registration and circulation of standardised TAMs. In recognition of their importance, heads of states and governments in Africa declared 2001–10 as the ‘decade of TAM’ during a meeting in Lusaka, Zambia [12], and 2011–20’ as the ‘second decade of TAM’ during a meeting in Windhoek, Namibia [13]. Since then, the WHO AFRO Member States (MS) have implemented the activities planned during these two decades through their plans of action on TAMs.

Through this literature review, we sought to synthesise evidence on the initiatives of WHO AFRO MS to standardise herbal medicines, or more precisely, to improve the

 regulation, administration, registration, and research capacity of TAM products. We aimed to encompass all information on policy, legal, and regulatory frameworks (national TAM policies, legal frameworks, regulation, legislation, and expert committees); the establishment of TAM structures in the MS’ ministries of health to coordinate the implementation of TAM activities (*e.g.* programmes, directorates, units, and national TAM Offices); the registration of TAM products (registration, market surveillance, and inclusion of TAM products in the national essential medicines lists); and research (establishment of institutes of TM Research and herbal pharmacopoeia, and presence of research institutes and training programmes) in a country-by-country analysis for the 47 WHO AFRO MS.

## METHODS

We searched Google and Google Scholar for relevant publications, as most of our information of interest is published in non-academic sources such as reports and webpages of the WHO, governments, and non-governmental organisations. This was done using the search string 'Herbal medicine” OR “traditional medicine” AND “country,” where the “country” was the specific name of a WHO AFRO MS. As an immediate former advisor of WHO AFRO on TAM and a WHO staff member, KOMJ also physically searched the WHO AFRO archives and institutional memory for non-published information.

We screened all retrieved publications to identify those that presented any of the steps that the WHO AFRO MS have taken to standardise herbal medicines. We prioritised publications authored between 2013 and 2023, as they covered the 2015–20 period when the WHO AFRO Regional Director introduced the Transformational Agenda to accelerate WHO reform and 10 years since the development of the strategy entitled ‘Enhancing the Role of Traditional Medicine in Health Systems: A Strategy for the African Region’. We also considered materials published in earlier years when they provided useful additional information.

After this selection process (performed by DK and verified by KOMJ and KU), we extracted and narratively synthesised details of the regulation and policy formation and summarised information on the establishment of administrative structures, registration systems, and research institutes into a tabular format. KOMJ addressed informational gaps in the narrative and the table from in-house WHO documents and her institutional memory as a regional advisor on traditional medicine at WHO.

## RESULTS

**Figure 1:**
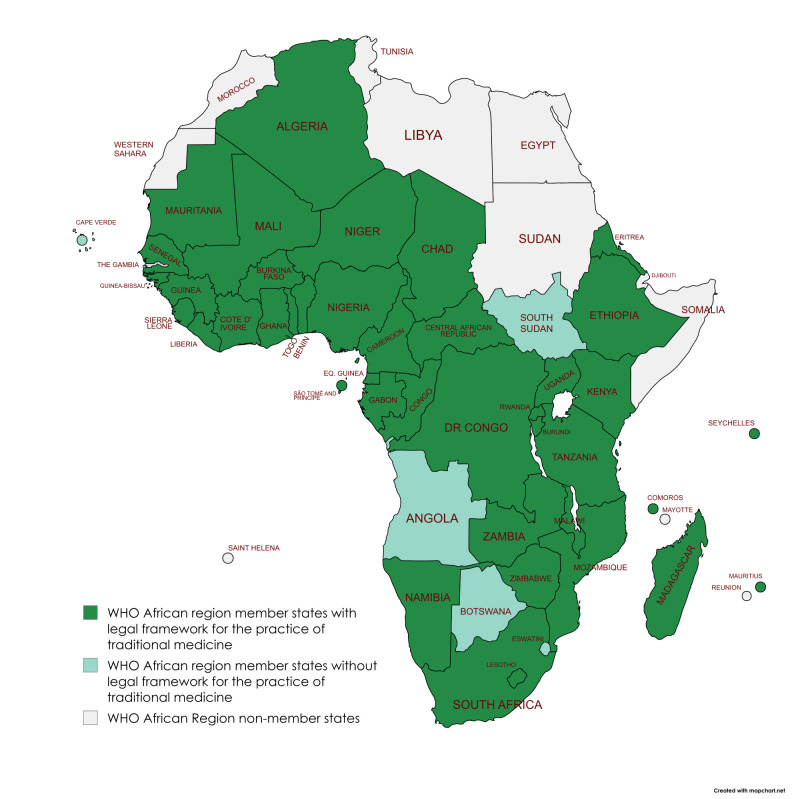
State of traditional medicine legislation in WHO AFRO MS based on literature review and published and non-published information available at the WHO AFRO Secretariat as of 2023.

The WHO AFRO MS achieved different milestones in the legislation, administration, registration, and establishment of research institutes for TM, with about 70% (33/47) of them having TM legislation (**Figure 1**, **Table 1**).

**Table 1:** Details of the establishment of administrative structures, registration systems, and research institutes in the WHO AFRO

Country	Traditional medicine structures in the MoH	Registration	Research and training
Algeria	A programme in the MoH coordinates TM activities, including the development of herbal medicines [14].	Assigning drug status to some herbal products might imply that they are being registered [15]. The National Center for Pharmacovigilance and Materiovigilance in the MoH monitors adverse events with the use of herbal medicines. It has provided an electronic template to be used in the reporting of adverse reactions by consumers of medicinal plants [16].	The Pharmacognosy and Api-phytotherapy research laboratory is in the Abdelhamid Ibn Badis University of Mostagem, which studies the activities of natural products [15].
Angola	The national programme on traditional and complementary medicine coordinates TM activities, and the National Office on TM are in charge of TM activities [17]. The National Department for Pharmacovigilance and Traditional Remedies is among the three departments in the National Directorate for Medicines and Equipment [16,17].	This literature review did not find evidence on the registration of herbal medicines in Angola.	The Angolan Herbal Pharmacopoeia is being developed [18]. The retrieved evidence did not show the existence of a research laboratory that focusses on herbal medicine research in Angola.
Benin	The TM programme and the National Office of TM in the MoH are in charge of TM.	Benin registers herbal medicines in line with WHO specifications (categories 1-4) [17,19] and requires market authorisation for categories 2–4 [20]. Herbal medicines feature in Benin’s list of essential medicines [21]. They are also included in the market surveillance system for the safety of medicines [17]. The approval of herbal medicines in Benin is regulated by a recent 2017 order [20,21].	Benin’s national research institutes focussing on herbal medicine include the Institute for Research and Experimentation in Medicine and Traditional Pharmacopoeia and the Laboratory of Pharmacognosy and Essential Oils in the Faculty of Sciences and Techniques at the University of Abomey-Calavi. Benin has also developed some monographs on medicinal and aromatic plants and pharmacopoeias to guide the development of quality herbal medicines. In addition, the country uses the West African Herbal Pharmacopoeia (WAHP) [21,22]. Benin has included TM in the curricula of health science studies.
Botswana	As of 2014, guidelines for registration of complementary or alternative medicine were issued by the Director of Clinical Services in the MoH [19].	The MoH and Wellness has a provision for the registration of complementary medicine. It has a form that applicants can fill out to comply with the registration requirements set by the Drug Regulatory Unit [21].	The University of Botswana has the Centre for Scientific Research, Indigenous Knowledge and Innovation that conducts research on herbal medicines [19]. The centre works with traditional health practitioners and researchers to advance herbal medicines in the country through knowledge development, innovation and entrepreneurship [21]..
Burkina Faso	The TM Programme and Pharmacopoeia and the National Office of TM in the MoH are in charge of TM.	Registration and market authorisation of herbal medicines are based on WHO guidelines; hence they feature the four categories of herbal medicines [17,20]. Burkina Faso’s national essential medicines list (NEML) includes several herbal medicines [17].	The Institute for Health Sciences Research established in 1984 and Pietro Annigoni Biomolecular Research Center in Labiogene, University of Ouagadougou established in 2006 conduct herbal medicine research. The MoH in Burkina Faso has developed some monographs; It has included TM in the curricula of health science studies [21].
Burundi	The National Programme on Traditional and Complementary Medicine and National Office on TM coordinate the implementation of TM activities [17].	Burundi does not have a registration system for herbal medicines [17].	The University of Burundi has a research institute - the University Research Centre in Pharmacopoeia and TM - that is dedicated to studying herbal medicines [17].
Cameroon	Herbal medicine in Cameroon is within the jurisdiction of the Ministry of Public Health. A subdirectory in the MoH focusses on traditional social-health care [23].	The Cameroonian Drug Regulatory Agency has taken significant steps toward the standardisation of herbal medicines, among them establishing a system of registering herbal medicines [23]. Its NEML includes herbal medicines [17].	Cameroon has a National Institute for Medical Research and Study of Traditional Medicinal Plants Studies established in 1974 that conducts research on herbal medicines [23]. The MoH of Cameroon has developed some monographs [24. Cameroon has included TM in the curricula of health science studies.
Cabo Verde	The TM Programme is established in the MoH.	A national regulatory authority registers conventional medicines but there is no information whether it registers herbal medicines.	There are reports of studies evaluating the toxicity, efficacy, and composition of herbal medicines in Cabo Verde [22].
Central African Republic	The Central African Republic has a Programme of TM and a National Office of TM in MoH.	Central African Republic has a system for registering herbal medicines. Although research evidence on the standardisation of herbal medicine in the country was not seen upon literature search, herbal products seemingly undergo value addition in the country since Hishimo Pharmaceuticals Pvt. Ltd identifies as a company in the Central African Republic that manufactures herbal medicine for the local market and export [17,25].	The University of Bangui has an African Pharmacopoeia and Traditional Medical Research Centre that conducts research on TM [17].
Chad	Chad has a TM Programme and a National Office of TM in the MoH.	Chad’s registration system for pharmaceuticals and the NLEM do not include herbal medicines.	The National Research Institute on TM and Herbal Medicine conducts research on TM. The University of N’Djamena hosts the Study and Research Unit in Pharmacopoeia and TM, which conducts research on herbal medicines [17]. Chad developed monographs on herbal medicines in 2013.
Comoros	The MoH has a TM unit [17].	Comoros does not have a system for registering herbal medicines [17].	The Comoran National Documentation and Scientific Research Centre conducts research on TM [17]. Institutions in Comoros are partnering with herbal medicine researchers to generate the evidence pivotal in standardising herbal medicines [26]. The Faculty of Science and Technology of the Comoros and Forest Research Centre partnered with the National Institute of Medicinal and Aromatic Plants (Morocco) to study the composition and biological activities of essential oils extracted from plants in Comoros. Scientists have attempted to characterise the phytochemical composition of traditional herbal products such as the Comorian tea, but they are yet to generate the data spectrum expected for standardisation [26].
Congo	The national office in the Congo is the TM Unit within the MoH and Population.	Congo has registered two herbal medicines used for the treatment of diseases of the digestive system, gynaecological disorders, hypertension, malaria, and amoebiasis, but has not yet included them in the NEML.	Researchers at the University of Marien Ngouabi in Congo have extensively studied the ethnobiology of medicinal plants and the anti-hypertensive, antibacterial, and antifungal effects of extracts of several medicinal plants.
Côte D'Ivoire	The government, through the MoH and Public Hygiene, has the National Programme for the Promotion of TM [17].	Côte d'Ivoire registers herbal medicines, but it has not yet included them in the NEML [27].	Researchers in Côte d'Ivoire and the Pasteur Research Institute-Institut Pasteur Côte d'Ivoire have conducted research on herbal medicines used for the treatment of diabetes and hypertension [28]. In 2018, the MoH of Côte d’Ivoire published a National Herbal Pharmacopoiea. The country has extensively studied the ethnobiology, phytochemical composition, antibacterial, antifungal, and anti-hypertensive among other in-vitro effects of medicinal plants’ extracts. The country has included TM in the curricula of health science studies.
Democratic Republic of Congo	Democratic Republic of Congo has both a Programme of TM and a National Office of TM in the MoH.	Several herbal medicines are registered, and some of them are included in its NEML [17].	The Institute for Health Sciences Research; Research Centre for Phytotherapy, African Pharmacopoeia and Pharmaceutical Technology; The Luozi Pharmaceutical Research Centre; School of Public Health, University of Kinshasa; and the National Institute of Biomedical Research conduct research on herbal medicines. The country has a pharmacopoeia for herbal medicines [17]. It has included TM in the curricula of health science studies.
Equatorial Guinea	Law 4/1985 created the National Directorate of TM in the MoH and Social Welfare [29]. Equatorial Guinea also has a National Office for TM.	Equatorial Guinea has a registration system for herbal medicines [17].	Equatorial Guinea has a national research institute for TM [17].
Eritrea	There is a TM unit through which the National Medicines and Food Administration of the MoH has been striving to regulate TM [30]. It also has a National Office of TM.	The Eritrean National Medicines Policy calls for the registration and regulation of marketed TMs [31]. It developed a Green form for reporting adverse events related to TM and endorsed the TM policy with support from the WHO [32]. Eritrea’s NEML and market surveillance system do not include herbal medicines.	The University of Asmara has a Medical Plants and Drug Discovery Research Centre that researches on TM in Eritrea [17]. In addition, the Department of Pharmaceutical Services in Eritrea is exploring approaches to analyse TMs toward standardisation [33].
Eswatini	Information on the existence of an office or a committee of experts for TM in the MoH was unavailable.	Retrieved information does not indicate the existence of a system for the registration of herbal medicines, but its pharmacovigilance policy includes herbal medicines by recognising that scientific evidence is needed to ascertain the safety of herbal medicines [34].	The University of Eswatini has established the Eswatini Institute for Research in TM, medicinal, and indigenous food plants to promote the use of TM in the national health system by generating scientific evidence [35].
Ethiopia	The Ethiopian Food and Drug Administration and the MoH provide direction on the practice of herbal medicine [36]. The Food, Medicine and Health Care Administration and Control Authority of Ethiopia is the National Office for TM [17].	Ethiopia has a registration system for herbal medicines [16]. Its NEML and market surveillance systems do not include herbal medicines.	The Ethiopian Health and Nutrition Research Institute in the Drug Research Department conducts research on TM in Ethiopia [17]. The MoH of Ethiopia has developed some monographs, and the country has included TM in the curricula of health science studies.
Gabon	Gabon’s MoH has a national office for TM [17] and a national programme on TM.	It is not clear from the information in the literature whether Gabon registers herbal medicines, but TM is among the components of the national health system [37].	The Institute of Pharmacopoeia and TM (IPHAMETRA) conducts research on TM used for the treatment of malaria, opportunistic infections related to HIV/AIDS, diabetes, and hypertension [38].
The Gambia	A national programme for TM was established in 2001, and a related national technical working group was formed in 2002 [17]. There is a National Office on TM.	The registration of herbal medicines manufactured for commercial purposes is done following the Medicines and Related Products Act of 2014 [39].	Based on the retrieved information, there is no research institute for the study of herbal medicines, although the MoH has developed some monographs.
Ghana	The Traditional and Alternative Medicine Directorate in the MoH has been coordinating traditional and alternative health systems since 1991 [40,41]. The country also has a National Office on TM [17].	The NEML in Ghana contains herbal medicines, with the Food and Drugs Board registering and carrying out market surveillance on herbal medicines [17].	The Center for Scientific Research into Plant Medicine in Ghana has been conducting research and promoting herbal medicine since 1975 [40]. The country uses the *Ghana Herbal Pharmacopoeia* (2nd ed., 2007) as its national pharmacopoeia, and *Ethnobotanical and floristic studies in Ghana* as the national monograph. As of 2012, 120 national monographs had been issued as part of the series titled ‘Monographs on medicinal plants of Ghana’. The country has included TM in the curricula of health science studies. Kwame Nkrumah University of Science and Technology in Ghana offers a four-year Bachelor of Science program in herbal medicine in its Department of Herbal Medicine [42].
Guinea	The MoH has a national programme on TM and a national office for TM.	The National Directorate of Pharmacies and Medicines is the national regulatory authority for registration of pharmaceuticals, including herbal medicines. The pharmaceutical regulatory law has given authority to the National Directorare of Pharmacies and Medicines for medicines’ quality assurance and surveillance of medicines in circulation, including herbal medicines.	The National Research Institute of Herbal Medicines conducts research on herbal medicines used for the treatment of malaria, opportunistic infections related to HIV/AIDS, hypertension, and COVID-19. Some researchers have initiated activities to establish a phytovigilance database that would facilitate the identification of toxic medicinal plants [43]. The MoH has published 18 national monographs of medicinal plants of Guinea. The country has included TM in the curricula of health science studies.
Guinea Bissau	The Directorate of Community Health Care Services and Promotion of TM in the MoH is the National Office for TM [17].	Information in the retrieved literature does not indicate the existence of a system for the registration of herbal medicines. The NEML does not contain herbal medicines [17].	Researchers in Guinea Bissau have started activities such as ethnobotanical studies and establishment of a database of medicinal plants to drive the country toward standardisation of TM [44]. In 2006, the coordinator of the National Secretariat to Fight HIV/AIDS in Guinea Bissau indicated prospects of funding TM research, but there are no signs of progress in this respect [45].
Kenya	The MoH of Kenya has a programme to coordinate TM activities in the country.	In January 2022, the Pharmacy and Poisons Board of Kenya published guidelines for the registration of herbal products [46]. Herbal medicines and products are identified among the health products and technologies to be regulated under the proposed Kenya Drug Authority Bill of 2022. The Authority will develop regulations for the commercial importation, manufacture, or sale of herbal medicines [47]. The manufacturers, suppliers, and sellers of herbal medicines will be licensed under the proposed Act [47]. However, legislation and policy on herbal medicine in Kenya are stuck at the draft stage [48].	Kenya has established research institutes for TM with the Center for TM and Drug Research at Kenya Medical Research Institute [49] and the National Phytotherapeutics Research Center at Kenyatta University [50] conducting research on the safety, efficacy, and quality of herbal medicines. The Health Act indicates that the Cabinet Secretary of the MoH shall make regulations for the documentation of TMs [51]. Universities including the University of Nairobi and Kenyatta University have postgraduate degree programs in pharmacognosy and alternative medicine with research projects on herbal medicines [52,53]. Kenya has included TM in the curricula of health science studies.
Lesotho	Lesotho has a national office of TM in the MoH.	Non-governmental organisations are the main custodians of TM in Lesotho [54]. Tens of commercial medicinal products mainly developed from plants are available in Lesotho, but it is not clear whether they are standardised and registered [55].	This literature review did not find information on the presence of a research institute for TM in Lesotho.
Liberia	The MoH and Social Work has a complementary medicine division [20]. A National Board of Complementary Medicine was established by an Act of Parliament [56]. There is a national office of TM in the MoH.	The Liberia Medicines and Health Products Regulatory Authority registers and regulates medicines, including herbal medicines [57]. However, the market surveillance system does not monitor herbal medicines [17].	This review did not find information on the existence of a research institute dedicated to TM in Liberia. Notably, Liberia hosted the 15th Ordinary Session of the Assembly of Economic Community of West African States Health Ministers in April 2014, which adopted the first West African Pharmacopoeia of Herbal Medicines published by the West African Health Organization [20].
Madagascar	Madagascar has a TM programme in the MoH for coordinating the implementation of TM work plans. It also has a national office of TM in the MoH. The Public Health Ministry has a Pharmacopoeia and TM Service to professionalise herbal medicine [58].	The NEML in Madagascar contains herbal medicines (16). The *Towards a Malagasy pharmacopoeia* monograph provides the manufacturing information for herbal medicines, which shows a commitment to registering herbal medicines [16].	Monographs of medicinal plants in some areas in Madagascar have been published [59]. Research institutions such as *Institut Malgache de Recherches Appliquées* and National Center for Applied Pharmaceutical Research and Homeopharma conduct research on natural product development [60]. The establishment of a manufacturing facility (Pharmalagasy) during the COVID-19 pandemic improved the infrastructure for the production of herbal medicines in the country [61]. Madagascar has included TM in the curricula of health science studies.
Malawi	There is a TM Programme in the MoH which also supports TM through the Malawi Traditional Healers Umbrella Organization [62].	Malawi has a system of registering TM through the Pharmacy and Poisons Board [62,63].	This literature review did not retrieve information on the existence of a research institute dedicated to herbal medicine in Malawi.
Mali	The National Institute of Research in the MoH has a Department of TM that was upgraded in December 2023 to an Institute of TM Research and Pharmacopoeia, which focusses on TM standardisation [58]. This institute also acts as the national office of TM in Mali.	Mali promotes the production of categories 3 and 4 herbal medicines based on the WHO criteria, which are improved and supported by scientific evidence [64]. The National Commission for Marketing Authorization in the MoH issue approvals based on the assessment of dossiers on the safety and efficacy of improved herbal medicines. The country’s list of essential medicines includes herbal medicines.	The Institute of TM Research and Pharmacopoeia in the MoH conducts research on the pharmacognosy of medicinal plants. It also supervises and examines Malian and international postgraduate students researching herbal medicine. The Ministry has also developed some monographs. It has included TM in the curricula of health science studies.
Mauritania	There is a TM programme and a national office of TM in the MoH. According to AUDA-NEPAD, the Directorate of Pharmacy and Laboratories in the MoH in Mauritania is in charge of promoting the improvement of TM [65,66].	This literature review did not find information on the registration of TM in the country.	This literature review did not find information on research on TM in Mauritania.
Mauritius	Although it is not clear whether Mauritius has a national office for TM, the website of the MoH and Wellness describes TM as among the hospital services provided in the country [64].	The Ayurvedic committee evaluates TM by verifying that they meet specific requirements on safety, efficacy, and quality for registration by the national regulatory authority [67]. The government imports Ayurvedic medicines from India and distributes them to government Ayurvedic facilities where the public can access them free of charge [67].	The University of Mauritius conducts research on TM used for the treatment of malaria, diabetes and hypertension. In addition, there is a research centre through which the country collaborates with India, which also offers scholarships for Mauritius’ citizens to study Ayurvedic medicine in India [68]. A two-volume publication of monographs on medicinal plants found on the island exists. TM is included in the curricula of health science studies.
Mozambique	The MoH created the Institute for TM in 2010 to coordinate the implementation of TM activities and improve TM. There is a national office of TM in the MoH.	The national regulatory authority of Mozambique registers herbal medicines based on the same criteria as conventional pharmaceuticals, following the national monographs, African Union Herbal Pharmacopoeia, and other pharmacopoeias. The herbal medicines are evaluated based on the WHO standard. They are, however, not included in the NEML [17].	The Ethnobotanical Development Center under the Ministry of Science and Technology was established in 2008 to research medicinal plants [69]. A TM Institute under the MoH also researches on herbal medicines [17]. Mozambique has included TM in the curricula of health science studies.
Namibia	The Department of Primary Healthcare in the MoH and Social Services has a TM program that coordinates all activities related to TM. The MoH also has a national office of TM. However, its activities have mainly been preparing for and holding the African TM day [70].	Although the reviewed literature did not indicate whether Namibia registers herbal medicines, the market surveillance system for safety of medicines in Namibia includes herbal medicines [70].	The University of Namibia conducts research on TM [17].
Niger	In 2018, Niger was in the process of developing a national programme for TM [17]. There is a Directorate of Pharmacy, Laboratories and TMs within the Ministry of Public Health [71,72]. The directorate implements policies, monitors activities, and participates in research to improve TM in Niger [72]. The National Office for Traditional and Complementary Medicine is within the MoH.	There is a system for registering TM and its NEML includes herbal medicines. Herbal medicines are regulated under the national pharmaceutical legislation in the non-prescription medicines category [17].	This literature review did not find information on the existence of a research institute for herbal medicines in Niger. TM is included in the curricula of health science studies in the country.
Nigeria	The Department of Traditional and Complementary and Alternative Medicine, which was created in the Federal MoH as a national office for TM, supervises herbal medicines in Nigeria. Nigeria has departments of TM in all 36 States of the Federation.	Nigeria has developed standardised herbal medicines for the management of various diseases. The products are registered by the National Agency for Food and Drugs Administration (NAFDAC), which validates herbal medicines for safety and therapeutic efficacy [73]. It also conducts pharmacovigilance on them [74].	Nigeria has research institutes such as the National Institute for Pharmaceutical Research and Development, Nigeria Natural Medicine Development Agency, and Research Institute of Traditional and Alternative Medicine that research, develop, and promote herbal medicines [73-76]. Several Universities in the country run undergraduate and postgraduate programmes in pharmacognosy and TM. Nigeria, which has a National Herbal Pharmacopeia published in 2008, has included TM in the curricula of health science studies.
Rwanda	Rwanda has a national TM programme and a national office of TM in the MoH [77.	The Rwanda Food and Drugs Authority registers herbal medicines using regulations that apply to all medicinal products [17]. The herbal medicines are not included in the list of essential medicines [78].	Research and training: The National University of Rwanda has a University Center for Research in Pharmacopoeia and TM that was transferred to the Institute of Scientific and Technological Research and later the National Industrial Research and Development Agency in 2013 [78,79]. The Centre for Research in Phytomedicines and Life Sciences in the Institute of Scientific and Technological Research has developed several botanical drug products. TM is part of the curricula of health science studies in the country.
São Tomé and Principe	There is a national office for TM in the MoH [17].	This literature review did not find information on the registration of TM products in the country.	There is no dedicated research institute for TM research. Medicinal Plants and TM of São Tomé and Principe are available in the repository of the Center of African Studies of the University Institute of Lisbon, Maria do Céu Madureira.
Senegal	A national programme for TM, which was preceded by a traditional pharmacopoeia office, has existed in the MoH since 1995. The national office for TM is in the Private, Occupational and TM Division of the Health Directorate, administered under the MoH.	Senegal has a national commission and a technical committee for registering natural products [17]. The procedures for registration of herbal medicines are simpler than conventional medicines, although the criteria for registration is the same [17]. Nevertheless, the commonly used herbal medicines in Senegal are not standardised [80].	PROMETRA, a Senegal-headquartered international non-governmental organisation that preserves and restores TM [81], conducts TM research at Experimental Center for TM at Fatik. Dr Yvette Parès (1926-2010) established the traditional hospital of Keur Massar in 1960, which was a major step toward standardisation of herbal medicine [82,83]. Senegal has included TM in the curricula of health science studies.
Seychelles	Seychelles has established the Complementary Health Care Services Board in its MoH [84].	It is not clear from the retrieved literature whether Seychelles has a system for registering herbal medicines. Some herbalists have progressed from home-based practice to established herbal stores, but there is no evidence that they have standardised their herbal medicines [84].	This literature review did not find information regarding herbal medicines research in Seychelles.
Sierra Leone	The MoH has a TM programme that has established a school, healing centre, and workshops for TM [85,86]. There is a national office of TM in the MoH.	The available literature had no information regarding herbal medicines registration in Sierra Leone.	This literature review did not find information regarding the existence of TM research institutes in Sierra Leone. Sierra Leone has included TM in the curricula of health science studies.
South Africa	The Department of Health has a directorate that coordinates and manages TM in South Africa. There is also a national office of TM in the MoH.	Information in the literature does not indicate whether the country registers TM products. Nevertheless, the government has provided tools to protect TM knowledge and the related intellectual property rights [87]. It has also issued market authorisations for TMs.	South Africa has a TM research institute and postgraduate programs in herbal medicine [88]. It has included TM in the curricula of health science studies.
South Sudan	TM does not feature in the organogram of the MoH of South Sudan [89].	Although the reviewed literature did not indicate the existence of a registration system for herbal products, traditional Chinese medicine is used at the Juba Teaching Hospital [90], which is a referral hospital in South Sudan.	The retrieved literature did not show that a research institute dedicated to TM exists in the country. Traditional Chinese medicine doctors offer training to medical students in South Sudan in efforts to promote the use of TCM [91,92].
Togo	Togo has a national TM programme and a national office of TM in the MoH.	This literature review did not find information on the existence of a system for registering TM products.	Togo has a Center for Studies and Research in Applied TM [93]. The Laboratories and Genetic Engineering Industries Annexes also conduct research on TM. It has included TM in the curricula of health science studies.
Uganda	There is a national TM programme and a national office of TM in the MoH.	TMs are approved using the same criteria as other medicines by the National Drugs Authority of Uganda. In 2023, the National Drugs Authority published the ‘Professional Guidelines on Registration of Imported Herbal Medicine Products for Human or Veterinary Use in Uganda’, which set forth guidelines for the preparation of product dossiers for imported herbal medicine products [94].	The Ugandan government funds research studies for the development and standardisation of herbal medicines [95]. The National Chemotherapeutics Research Laboratory in the MoH established by the Uganda National Health Research Organisation conducts herbal products analysis to generate the safety and quality data needed for registration by the National Drugs Authority [96]. Other research institutes that conduct TM research in Uganda include the Lung Institute, Makerere University, and the Pharm-Biotechnology and TM Center, Mbarara University of Science and Technology. Uganda has included TM in the curricula of health science studies. In 2020, the National Drugs Authority launched the ‘Professional Guidelines on Clinical Research on Herbal Medicine Products’ to ensure clinical research into herbal medicinal products is conducted according to national and international ethical standards, adheres to recognised, credible procedures subject to methodical documentation, and protects the interests of the researchers and the rights and safety of patients [85]
The United Republic of Tanzania	The MoH and Social Welfare hosts a National TM Programme and a National Office of TM in the MoH.	In 2014, the Tanzania Food and Drugs Authority published guidelines for the registration of traditional medicinal products [97]. The guidelines specify the safety, quality, and effectiveness requirements for registration of herbal products according to the Materia Medica Regulations [16]. The NEML and market surveillance system for the safety of medicines do not include herbal medicines.	The Institute of TM at the Muhimbili University of Health and Allied Sciences conducts short courses to empower traditional health practitioners to develop TMs. It has also developed, patented, and registered herbal products, which it sells to the public [98]. The National Institute for Medical Research also conducts TM research [16].
Zambia	This literature review did not find the existence of a national office on TM in the MoH of Zambia.	The Pharmaceutical Regulatory Authority in Zambia, which published the guidelines for the registration of TM in 2023 [99], has registered some herbal medicines [16]. The Zambian government is allowing the establishment of traditional Chinese medicine in the country while initiating collaborations to leverage the Chinese expertise and technology to standardise TM in Zambia [100].	The National Institute on Medical Research, Tropical Diseases Research Centre; The Malaria Institute at Macha, and The Zambia Institute for Research in Traditional Remedies conduct research on TM [17,101]. The MoH and the National Health Research Authority in Zambia collaborated to publish guidelines for research in traditional, complementary, and alternative medicine in Zambia in 2018 [102]. Zambia has included TM in the curricula of health science studies.
Zimbabwe	The MoH and Child Care has a TM unit in the department of curative services [91].	In 2010, the Zimbabwean parliamentary health committee recommended that the government should create a regulatory framework for standardisation of herbal medicines through manufacturing and patenting, but there is no information in the literature whether this has been done [92]. The Medicines Control Authority of Zimbabwe has published guidelines to facilitate the registration of complementary medicines [93].	The National Research Institute (Blair) and the University of Zimbabwe conduct TM research and development [27]. Zimbabwe has included TM in the curricula of health science studies.

### Algeria

The Consumer Protection and Fraud Repression Law No. 09-03 is commonly applied to regulate TAM products, which are considered foodstuff. Product safety and consumer information are regulated according to conditions outlined in the decrees no. 12-203 and 13-378, respectively. Products with preventive or curative properties are considered drugs, hence they are regulated under Health Law No. 18-11 [13]. Herbal medicines are regulated by national regulatory authority under the Ministry of Health (MoH) following a simplified authorisation process [13]. Information in the retrieved literature does not point to the existence of a national policy on TM or the inclusion of objectives on TAM in the national drug policy in Algeria. The country’s MoH has a TAM programme and a pharmacognosy and api-phytotherapy research laboratory, but there is no evidence that it registers herbal medicines [14–16,103].

### Angola

In August 2020, Angola’s council of ministers approved the national policy on TM to guide the research on and technological development of herbal medicines toward their integration into the national health system [18]. However, we could not find information on whether the country has a related law or regulations. Although Angola has a national programme, office, and department in the MoH, it neither has a registration system nor a dedicated research institute for TM [16,17].

### Benin

Benin’s National Policy on Pharmacopoeia and TM provides the basis for developing herbal medicine in Benin [16]. The country’s legislation on the regulation of herbal medicine is outlined in Order No. 2017-017/MS/DC/SGM/CTJ/DPMED/DA/SA016SGG17, while its National Committee for the Regulation of TM started functioning in 2017 [104]. There is a National Expert Committee for TM. Benin also has a programme, national office, registration system, and research institutes for TM [17,20,27,104].

### Botswana

Botswana has a national policy, but has not established dedicated legislation for TM [16]. However, it is in the process of passing a law to govern TM [19,21], and has an expert committee on TM. The country has a centre within a university that conducts herbal medicine research and a registration system for TM, but lacks a dedicated programme or office [19,21].

### Burkina Faso

Burkina Faso has a national policy on herbal medicine, titled ‘National Policy Framework related to TM and Pharmacopoeia’, and also includes it in national health policies and strategic plans. The national laws regulating herbal medicine are part of the Public Health Code (No. 23/94/ADP) [22]. The 14 December 2004 Decree N°2004-569/PRES/ PM/MS/MCPEA/MECV/MESSRS, meanwhile, regulates herbal medicines [104]. The country also has a TM programme, office, registration system, and research institutes [17,20].

### Burundi

Burundi has a national policy for the practice of TM. The Decree 100/253/2014 regulates the use of medicinal plants, specifically banning the use of secret remedies or remedies not registered in the national list. The enforcement of the decree is weak, and few organisational and financial measures have been taken to implement its prescriptions [105]. Burundi has a national programme and office for TM and a TM research institute, but lacks a registration system for herbal medicines [16].

### Cabo Verde

TM is included in national health policies and strategic plans in Cabo Verde. The country has a national expert committee for TM and a TM programme, but there is no evidence in the literature that it registers herbal medicines. Although studies on the evaluation of herbal medicines exist in the country, it is not clear whether it has a research institute dedicated to TM [20].

### Cameroon

Cameroon’s national policy on traditional and complementary medicine is integrated into other national policies such as the Healthcare Sector Strategy 2001–2015, the Health Framework Law, and the TM Strategic Development Plan 2006–2010. The country has a national expert committee, programme, office, registration system, and research institute for TM [16,20,23,24].

### Central African Republic

The Central African Republic has a national policy titled ‘Central African Republic National Policy on TM’ and a law for the practice of TM. It also has several decrees and orders to govern the organisation of TM, but there are no regulations in place for the manufacture of herbal medicines. A ministerial order forms a temporary expert committee on TM whenever a need arises [16]. The Central African Republic has a national programme, office, registration system, and a research centre for TM [17,25].

### Chad

Chad has a national policy on TM and has included TM in its national health policy and strategic plan. It has a legal framework for the practice of TM and a national expert committee for traditional and complementary medicine [17,27], as well as a national TM programme, office, registration system, and research institute [16].

### Comoros

Comoros has a national policy to govern TM, a legal framework for the practice of TM, and a TM law [16]. Besides, TM is included in its national health policies and strategic plans. The country also has a TM unit in the MoH and a TM research centre [26], but lacks a TM registration system [17].

### Republic of the Congo

The Republic of the Congo has a national policy on TM and a legal framework for the practice of TM. It is included in the national health policy and strategic plan. Congo has a TM unit in MoH and a TM registration system, but lacks a research institute dedicated to TM studies [17].

### Côte d'Ivoire

Côte d'Ivoire has a national policy on TM, a TM law, and a decree to govern the organisation of TM [16]. TM is also included in national health policies and national health strategic plans. Côte d'Ivoire also has a national programme and a registration system for TM. Although the country conducts research on herbal medicines [28], there is no evidence on the existence of a research institute dedicated to TM [27,28].

### Democratic Republic of Congo

The Democratic Republic of Congo has a national policy on TM [16] and Law No. 18/035 on TM issued on 13 December 2018. The country has included TM in its national health policy and strategic plans, and has an expert committee, a national programme, registration system, and research centres for TM [16].

### Equatorial Guinea

Equatorial Guinea has a national policy for TM and a law governing the practice of TM. It has a directorate of TM in the MoH, a registration system, and a TM research institute [17,29].

### Eritrea

Eritrea has a national policy on TM and a legal framework for the practice of TM. It also has a national advisory committee on TM [16], and a TM unit, national office, registration system, and research centre [30-33].

### Eswatini

Although there have been advanced discussions on the integration of TM with modern medicine, Eswatini is yet to establish any related legislation and structures [106]. It lacks a national office and a registration system for TM, but has a dedicated research institute [34,35].

### Ethiopia

By December 2020, Ethiopia did not have independent regulation to govern herbal medicine [36]. The national health, medicine, and science and technology policies were reflected in Proclamation 1112/2019 to guide the use of herbal medicines in Ethiopia [17]. The country has a national office, registration system, and a research institute for TM [16].

### Gabon

Gabon has a national policy on and a legal framework for the practice of TM, as well as a national expert committee on TM. The country included TM in its national health policies and strategic plans, and also has a national office, national programme, and a research institute for TM, but it is not clear whether it has a registration system for TM [17,37,38].

### The Gambia

The Gambia has a national policy on TM, although it does not have related legislation [17,39]. It also has a national expert committee, a national programme, technical working group, national office, and a registration system for TM, but lacks a dedicated research institute [17,39].

### Ghana

Ghana has a national policy, legislation, and a national expert committee on TM, which is included in its national health policies and strategic plans. Its MoH has created policies and institutions for TM [40]. The country has a TM directorate, national office, registration system, and research centre [17,41,42].

### Guinea

Guinea has a national policy on TM and a legal framework for the practice of TM, and has included it in its national health policies and strategic plans. It has a national expert committee, national programme, national office, a registration system, and a research institute for TM [43].

### Guinea Bissau

Guinea-Bissau has regulated TM through national policy and regulation since 2010 [16]. It has a national office for TM, but there are no indications that it has a dedicated research institute and a registration system [17,44,45].

### Kenya

Kenya has a national policy on TM and medicinal plants, and has included TM in its national health policies and strategic plans. The MoH, through the Division of Traditional and Alternative Medicine, has drafted a legal framework for the practice of TM titled ‘A Traditional and Alternative Health Practitioners Bill 2020’. Furthermore, part X of the Health Act of 2017 specifically addresses traditional and alternative medicine [46–48,78] and outlines that the MoH shall develop policies to guide the standardisation of TM. The Act provides for the establishment of a regulatory body for traditional and alternative medicine (which is yet to be established) through an Act of Parliament. The country has a national expert committee, a national programme, registration system, and research institutes for TM [49,50,52,53].

### Lesotho

While Lesotho has a legal framework for the practice of TM, the literature provides no evidence of a system for the regulation of herbal products in the country [107]. While Lesotho has a national office for TM, we found no proof on the existence of a dedicated registration system and research institute [54,55].

### Liberia

Liberia developed the national policy and strategy on TM in 2015 with commitments to support the standardisation of herbal medicine [16]. It has a legal framework for the practice of TM and a national expert committee, a national division, board, and registration system, but lacks a dedicated research institute [27,56,57].

### Madagascar

Madagascar has a national policy for TM, a legal framework for the practice thereof, and a national expert committee. Its MoH recognised TM as legitimate in 2007 [59]. The country also has a national programme, office, service, registration system, and a research institute for TM [17,60,61].

### Malawi

Malawi has a national policy on and a legal framework for the practice of TM, as well as a dedicated national expert committee. In 2018, its Government expressed intentions to formulate a national agenda for TM and start regulating them in collaboration with the Pharmacy and Poisons Board. Malawi also has a national programme and a registration system for TM, but it lacks a dedicated research institute [62,63].

### Mali

Mali has a national policy on TM, a legal framework, and a decree that fosters good manufacturing practices for herbal medicines. It also has a national expert committee, a department, national office, registration system, and a research institute for TM [58,64].

### Mauritania

Mauritania has a national policy on TM and a legal framework for its practice. It is also included in national health policies and strategic plans. The country has a national expert committee for TM and a national programme, but it neither has a dedicated research institute nor a registration system [65,66].

### Mauritius

Mauritius included TM in its national health policies and strategic plans. It formulated and promulgated the Ayurvedic and Other TMs Act of 1990 to aid the recognition of the Ayurvedic healing that is practiced by the Hindu-Mauritian population [68,108]. Furthermore, TM is included in public health care, but is yet to be regulated by a dedicated law [67]. An Ayurvedic committee exists under the MoH and Quality of Life [108]. Mauritius does not have a national office and a research institute for TM; it has a system for registering Ayurveda medicines [64,67,68].

### Mozambique

Mozambique governs TM through a policy and strategy issued in 2009 [16]. It has a legal framework for the practice of TM, and has included it in its national health policies and strategic plans. The country established a national expert committee for TM in 2012 for the purpose of legislation and regulations. It has a national institute, office, registration system, and a dedicated research centre for TM [17,109,110].

### Namibia

Namibia has a national policy on TM and a legal framework for its practice, entitled ‘Access to Biological and Genetic Resources and Associated Traditional Knowledge Act 2 of 2017’ [70]. It also included TM in its national health policies and strategic plans. The country has a national expert committee for TM, and a national programme, office, and registration system for TM, but likely lacks a dedicated research institute [17,70].

### Niger

Niger has a national policy on TM, has included it in its national health policies and strategic plans, and has been implementing a national strategy since 2002. It has a national office, directorate, and a registration system for TM, but lacks a dedicated research institute [17,71,72].

### Nigeria

Nigeria has a national policy on TM and has included it in the national health policy and strategic plan, with some states formalising TM by enacting regulations and establishing regulatory boards [73]. Nigeria also has a national expert committee on TM, a national department and separate state departments, national office, registration system, and dedicated research institutes [74–76].

### Rwanda

Rwanda has a national policy on traditional, complementary, and alternative medicine [77,79], and has included TM specifically in national health policy and strategic plan. Herbal products are subjected to the same regulations as other medicinal products (Rwanda FDA Law No 003/2018 of 09/02/2018, Article 9) [77]. There is a national expert committee on TM, and a national programme, office, registration system, and research centres for TM [17,78].

### São Tomé and Príncipe

In São Tomé and Príncipe, herbal medicine is governed by the national programme for essential drugs and TM [17,69]. The country has a national office for TM, but lacks a registration system and a dedicated research institute.

### Senegal

Senegal’s national policy for TM is integrated into the Strategic Plan for Promotion of TM in the Health Care System 2007-2010 [89]. In 2017, a legislative proposal on TM was adopted by the Ministerial Council and introduced in the Senegalese parliament but it is yet to be implemented due to opposition from some stakeholders [89]. There is a national expert committee on TM. Senegal has a national programme, office, division, and a registration system for TM, but it lacks a TM research institute [17,80–83,89].

### Seychelles

Seychelles has a legal framework for TM. It has a national board for TM but there is no evidence that it has a TM research institute and a registration system for TM [84].

### Sierra Leone

The ‘National Health Sector Strategic Plan (2021–2025)’ by the MoH and Sanitation of the Government of Sierra Leone states that TMs and products ought to be integrated into the formal sector through research and development [85]. There is a national expert committee on TM, and a national programme and office for TM, but there is no dedicated registration system or research institute [86].

### South Africa

South Africa has a national policy on TM and has included TM in its national health policy and strategic plan. The ‘Traditional Health Practitioners’ Act of 2007’ regulates TM [111]. The country also has a national expert committee on TM, and a national directorate, office, and a dedicated research institute for TM, but it is not clear whether it has a registration system [87,88].

### South Sudan

While South Sudan lacks a legislation, policy, national programme, national office, registration system, or research institute for TM, traditional Chinese medicine is practiced at Juba Teaching Hospital in South Sudan [90–92].

### Togo

Togo has a national policy on TM and has included it in its national health policy and strategic plan, but we found no evidence of any legislation. The country has a national expert committee, a national programme, office, and research centre for TM, but lacks a registration system [93].

### Uganda

The first substantive governance of the traditional and complementary medicine sector is embedded within Uganda’s 2005 ‘National Policy on Public Private Partnerships for Health’. The country passed the ‘Traditional and Complementary Medicines Act of 2019’ [78,94]. It has a national expert committee, a national programme, office, registration system, and research centres for TM [78,94–96].

### The United Republic of Tanzania

The United Republic of Tanzania has a policy for TM and has included it in its national health policy and strategic plan, while it is further governed through the ‘Traditional and Alternative Medicine Act’ [112]. The country has a national expert committee on TM, a national programme, a registration system, and a dedicated research institute [17,97,98].

### Zambia

Zambia has a national policy on TM and has included it in its national health policy and national health strategic plan. It has a legal framework for the practice of TM and a national expert committee on TM. While it lacks a dedicated national office, it has both a registration system and a research institute [17,99,100,102].

### Zimbabwe

Zimbabwe has a national policy on TM and has included it in its national health policy and national health strategic plan. It has a legal framework that recognises TM [113,114], a national expert committee, a TM unit, a system for registering complementary medicines, and a dedicated research institute [17,115,116].

## DISCUSSION

### Policy, legal and regulatory framework

Most WHO AFRO MS have developed policies on TM and included TM in the national health policies and strategic plans, which could be due to the high rate of the adaptation of WHO TAM tools to fit the local contexts [9]. For example, the number of MS with TM policies increased substantially from eight in 2000 to 40 in 2020, as did the number of MS with operational plans for policy implementation from only one to 28 in the same period [27]. Overall, MS have established the necessary policies to move towards the integration of TM into the mainstream healthcare system.

Most MS have developed legislation for governing the practice of TM, which is a sign of improvement since several MS did not have clear legislative criteria for TM by 1998 [117]. With the support of the WHO, several MS have strengthened regulatory systems for phytomedicines [101]. Thirty-nine out of 47 MS have already developed legal frameworks for the practice of TM and prepared bills of parliament that are in various stages of debate and approval before being enacted to become law. Burkina Faso and Mali are role models in the legislation of herbal products, since they have specific decrees for herbal products besides the regulatory framework for TM practice. However, a few MS are slow in passing and assenting to the legislation to govern TM. Accordingly, MS with ongoing or proposed legislative activities, such as Kenya and Senegal, should fast-track the passing of the pending laws for the regulatory framework of herbal medicines to be well-established.

### Existence of TM structures in the MoHs

Most MS have established structures like national offices, directorates, and programmes in their MoHs to regulate TM. Only Botswana, Eswatini, South Sudan, and Zambia did not have such structures, while those in Seychelles and Mauritius were only partially dedicated to TM. This agrees with a previous report that MS with TM structures in MoHs for coordinating TM activities increased from 10 in 2000 to 38 in 2020 [27]. The existence of these structures indicates better prospects for the integration of TM into the mainstream healthcare system [101], since they provide avenues for the executive arms of government to implement TM policies and legislation.

### Registration

The majority of the MS registered herbal medicines using the systems for registering conventional drugs, with only a few including them in national essential medicines lists and pharmacovigilance programmes. Most national drug regulatory authorities have reviewed the pharmaceutical registration systems to include registration of herbal medicines based on the guidelines developed by the WHO AFRO [11]. Considering the unique nature of herbal medicines, there is a need for all MS to have a registration system dedicated to herbal medicines.

The MS that can serve as role models in the establishment of registration systems for herbal medicines and their inclusion in their national essential medicines list include Algeria, Benin, Burkina Faso, Cameroon, the Democratic Republic of Congo, Ghana, Madagascar, and Niger. Thus, the number of registered herbal medicines has increased from only 20 in 2000 to over 100 in 2022 [27]. Moreover, over 45 of the registered TM products were included in national essential medicines list during the same period. Significant huge room for improvement exists, however, as some MS were yet to start registering herbal medicines as of 2023.

### Research and training

The existence of herbal medicine research institutes and centres, monographs on medicinal plants, and the inclusion of herbal medicine content in school curriculums in most MS shows that they have applied the available tools and strategies to advance research and development. These efforts have resulted in the number of TAM-dedicated research institutes increasing from 18 in 2000 to 34 in 2020, and the number of MS with such institutes growing from 18 to 26 in the same period [27]. Role models for the establishment of research institutes include Benin, Burkina Faso, Chad, Kenya, and Madagascar. The MS can also learn about the establishment of pharmacopoeia from Angola, Cote D’Ivore, Democratic Republic of Congo, and Ghana, with the lattermost standing out in education and training since it offers a bachelor’s programme in herbal medicine.

Most of the TM research institutes are government agencies, while others are established within universities. They mostly conduct pre-clinical safety and efficacy testing, which are laboratory studies focussed on characterising herbal medicines. Others have progressed to product development through clinical trials toward registering WHO category 3 herbal medicines.

### Limitations

The information in this literature review may be incomplete, since some MS may have made non-published steps in the standardisation and registration of herbal medicines that were also absent in the institutional memories of the authors. Secondly, although Google and Google Scholar were the ideal search engines, since documentation of steps toward standardisation and registration of TM is more likely to be in grey literature, they are not exhaustive in their scope, so relevant literature might have been overlooked.

## CONCLUSIONS

Several WHO AFRO MS have made substantial progress in establishing systems for the standardisation, registration, and regulation of herbal medicines, with most having TM policies, legislation, structures in MoHs, registration systems for TM products, dedicated research institutes, and training facilities and curriculums integrating TM. Some MS like Benin, Burkina Faso, Ghana, and Madagascar have substantially progressed in instituting herbal medicine policies and systems, hence they are role models. Studies on the practical challenges in establishing and implementing policies and structures, as well as the shortcomings of WHO AFRO herbal medicine-related initiatives are recommended.
